# Factors related to dropout intention of medical college postgraduates in China: A comparison between students who receive standardized training and non-standardized training

**DOI:** 10.1371/journal.pone.0325146

**Published:** 2025-07-02

**Authors:** Haiyang Li, Jing Tong, Xue Jiang, Dan Gu, Xiaoning Zhang, Bin Hu

**Affiliations:** 1 School of Public Health, Xuzhou Medical University, Xuzhou, China; 2 School of Graduate, Xuzhou Medical University, Xuzhou, China; 3 Institute of Medical Humanities, Xuzhou Medical University, Xuzhou, China; 4 School of Stomatology, Xuzhou Medical University, Xuzhou, China; 5 School of Nursing, Hangzhou Normal University, Hangzhou, China; 6 Zhejiang Philosophy and Social Science Laboratory for Research in Early Development and Childcare, Hangzhou Normal University, Hangzhou, China; Universiti Sains Malaysia - Kampus Kesihatan, MALAYSIA

## Abstract

**Introduction:**

The global shortage of healthcare workers significantly impacts patient well-being and physician workforce development. Ensuring the graduation of every medical student is a critical way to maintain an adequate physician workforce. However, previous studies have shown that dropout intention is very high among medical college postgraduates in China. Therefore, there is a high need to investigate the factors associated with dropout intention among Chinese medical college postgraduates to prevent dropout intention and dropout rates.

**Methods:**

A cross-sectional study was conducted in December 2023 in Jiangsu Province, China. A total of 1042 postgraduates participated in the survey, with an effective response rate of 97.4%. The survey included postgraduates’ demographic characteristics, tutor-postgraduate interaction, research training environment, general psychological distress, and fear of future violence at work. The forward multivariate stepwise logistic regression was used to explore the factors related to dropout intention in medical college postgraduates who receive standardized training and non-standardized training, respectively.

**Results:**

We found that 16.5% of medical college postgraduates had an intention to drop out, with 13.0% among those in standardized training and 19.6% among those in non-standardized training. For the standardized training group, age, tutor-postgraduate interaction (professional ability interaction), and fear of future violence at work were risk factors for dropout intention, while a positive research training environment and high research self-efficacy were identified as protective factors. For the non-standardized training group, the father’s education and stress were risk factors, while satisfaction with the research degree program, tutor-postgraduate interaction (comprehensive cultivation interaction), and high research self-efficacy were protective factors.

**Conclusion:**

Factors influencing the dropout intention of medical college postgraduates in China vary between those who receive standardized training and non-standardized training. Chinese medical colleges and relevant education departments should develop personalized strategies for these two groups of students to reduce the dropout intention and dropout rate.

## Introduction

The pressing global shortage of healthcare workers poses a significant threat to patient well-being and the development of the medical workforce [1,2]. The World Health Organization’s projection of a staggering 12.9 million shortfall by 2035 underscores the urgency of this issue [3]. Moreover, the attrition of medical students exacerbates the shortage, emphasizing the criticality of ensuring the successful graduation of every student to maintain an adequate physician workforce [4–7]. China’s “14th Five-Year Plan” for healthcare personnel, while noting an increase in the total number, also stresses the need for high-quality development, aligning with the goal of fostering successful medical postgraduate graduations [8].

In China, medical degrees are clearly classified into three levels: bachelor’s, master’s, and doctoral. Most medical students require five years of undergraduate education and three years of graduate education to meet the academic requirements for positions in the healthcare system. However, data from China show that 13.6% of medical master’s degree students fail to graduate on time. Dropout has emerged as a complex and global concern in medical education, encompassing students who interrupt their school studies without completing their degree [9,10]. Previous studies have shown that the dropout rate of postgraduate medical students ranges from 5.0% to 12% [11–14]. Considering the high stakes of dropout for medical students, institutions, and society, growing studies have instead focused on dropout intention [15]. It was reported that 39.1% (1,383 individuals) of Chinese medical students had considered dropping out [7], 25.2% of 2222 U.S. medical students had dropout intentions, with 11% seriously considering dropping out each year [4]. European Commission’s Education and Training Monitor 2020 showed that 10.2% of students are early leavers from education and training [16]. The average attrition rate for 40 medical schools globally is 11.1% [10]. Dropout intention has far-reaching negative consequences for individuals, families, academia, and society at large [16]. Thus, it is imperative to delve into the factors that lead to the dropout intention among medical college postgraduates in China, which helps to prevent high dropout intention and dropout rates, alleviate the pressure of the shortage of healthcare personnel, and improve the quality of the healthcare workforce.

Previous research on factors associated with medical students’ dropout intention includes sociodemographic [17,18], interaction with supervisors [19,20], working environment [6], and mental health issues (i.e., burnout, depression, anxiety, stress) [4,21,22]. It is important to note that previous studies have focused on only one or a few of these influence factors. Furthermore, research on the dropout intention of Chinese medical students has predominantly focused on undergraduates or hospital interns, with limited attention given to the postgraduate cohort. Additionally, the data of few studies on the dropout intention of Chinese medical graduate students have mainly been published in China, which hampers information exchange and collaboration with international scholars. Therefore, it is necessary to conduct a comprehensive and in-depth investigation of the factors influencing the dropout intention of medical college postgraduates in China. Based on previous studies, this study comprehensively examined the factors influencing the dropout intention of medical college postgraduates in China from various perspectives: personal, interpersonal, environmental, and psychological.

Firstly, according to the Conceptual Schema for Dropout from College, individual characteristics of students prior to enrollment are important factors that influence the level of pre-enrollment goal commitments and institutional commitments, ultimately affecting dropout decisions [23]. For example, studies have found that demographic variables such as gender [24], grade level [25], family background (parents’ education and income levels) [18,26], and academic achievement [18] are associated with willingness to drop out of school. Additionally, academic satisfaction is a significant predictor of dropout intention [26,27].

Secondly, in China, the tutor system is utilized for postgraduate training. Tutors significantly influence the developmental process of students, promoting their research interests and career development [28–30]. Meanwhile, tutor-postgraduate interaction can play a significant role in postgraduates’ dropout intention [31]. Tutor-postgraduate interaction (TPI) is usually categorized into professional ability interactions and comprehensive cultivation interactions. The former involves scientific research and academic discussion, focusing on the teaching behavior of tutors toward postgraduate academic and clinical ability, the latter pertains to mentors’ support in postgraduate personal life and addressing life difficulties [32]. Inadequate advisor relationships were found to contribute to student attrition [33].

Thirdly, the importance of Research Training Environment (RTE) in medical and health professions education cannot be underestimated [34] and has a significant impact on medical students’ dropout [35]. The RTE theory suggests that a positive research training environment can increase graduate students’ interest in research and self-efficacy [36]. High levels of academic self-efficacy help postgraduates maintain more optimistic expectations about their research future, which can reduce their rejection of academic careers and increase their enthusiasm for engaging in research [37]. Conversely, bullying in the research environment negatively affects medical students’ sense of belonging and major satisfaction, which increases their intention to drop out [38].

Fourthly, Chinese medical college students exhibit a higher prevalence of poor mental health compared to their peers in other majors and medical students from other countries [39,40]. Studies have demonstrated a strong correlation between psychological issues (i.e., burnout, depression, anxiety, stress) and the risk of dropping out [4,16,21]. Research also indicates that healthcare workers experience higher rates of workplace violence [41], negatively impacting their mental health [42]. Additionally, healthcare workers may fear violence even if they have not experienced it directly [43]. This fear of future workplace violence, similar to its effect on healthcare workers’ career intention [44], may negatively influence the dropout intention of medical college graduate students.

Generally speaking, the aforementioned factors may affect medical college postgraduates’ dropout intention, however, these factors among medical college postgraduates in China may vary across different training modes. There are two training modes for medical postgraduates in China, which depend on the training contents and environment: standardized training (ST) and non-standardized training (NST). The former is guided by clinical practice, emphasizing the integration of medical theory and practice, with standardized residency training “Double Tracks in One”, with the ultimate goal of training compound clinicians with high quality. While, the latter is academically oriented, emphasizing fundamental theories, and primarily involves acquiring scientific experimental methods and techniques through laboratory settings, with the ultimate goal of cultivating scientific research talents [45]. Medical postgraduates’ perception of the healthcare environment and medical training significantly influence mental health and dropout risk [6]. To date, few studies have investigated the factors that influence the dropout intention of Chinese medical colleges for both ST and NST postgraduates. Therefore, this study was the first one aimed to explore factors influencing the dropout intention of medical postgraduates who receive ST and NST in China, respectively.

In sum, given the limited research on the dropout intention of postgraduates who received ST and NST in medical colleges in China, this study was conducted to fill this research gap. Specifically, the study aimed to investigate the factors related to dropout intention of Chinese medical college postgraduates who received ST and NST respectively, and explore their potential similarities and differences. Additionally, this study also aimed to characterize dropout intention rates for both groups, given that there is limited such data available and it also could be regarded as a data update.

## Materials and methods

### Participants and procedure

This cross-sectional survey was conducted using the convenience sampling method at Xuzhou Medical University in Jiangsu, China, from 01/12/2023 to 31/12/2023. The questionnaire survey was published through the Questionnaire Star network platform (www.wjx.cn). The respondents could fill in the questions via both their computer and their mobile phone. Our study did not rigidly restrict the answer time and did not emphasize speed but rather authenticity, which was used to minimize the impact of early delivery of responses by participants. Participants who completed the questionnaire in 5 minutes were excluded according to the pre-survey, as a result, a total of 1042 questionnaires were included in the final analysis (the effective response rate was 97.4%). All participants were informed of the contents, objectives, duration, risks, ethics approval, and data policy before providing informed consent, then they were allowed to proceed further in the questionnaire. All participants were informed of voluntary participation [46], and assured they could withdraw from the study at any time and their data would remain anonymous. The research was conducted following the guidelines of the Declaration of Helsinki and approved by the ethics committee of the Institutional Review Board at Xuzhou Medical University (reference number: XZHMU-2023117).

### Measurements

#### Postgraduates’ demographic characteristics.

In this study, nine demographic variables were collected, including (1) gender (male, female), (2) age (≤25, 26 ~ 30, ≥ 31), (3) grade (first-grade master, second-grade master, third-grade master), (4) academic performance (the first third, the middle third, the last third), (5) source of students (urban, rural), (6) one-child households (yes, no), (7) father’s education level and (8) mother’s education level (junior high school or below, senior high school technical secondary school, college or above), and (9) satisfaction with the experience of the research degree program. The last variable was measured based on a question: “Overall, are you satisfied with your experience of the research degree program?” Possible responses were Very dissatisfied/Not satisfied/General/Satisfied/Very satisfied.

#### Tutor-postgraduate interaction.

Tutor-postgraduate interaction was measured by using the Chinese medical college tutors-postgraduates’ interaction scales, which were designed by Harbin Medical University [32]. The questionnaire has 14 questions and includes two subscales: professional ability interaction (seven items, i.e., “The tutor is very strict with your scientific research”) and comprehensive cultivation interaction (seven items, i.e., “The tutor often chats with you”). Responses are made on a 5-point Likert scale, ranging from 1 = very inconsistent to 5 = very consistent. The subscales’ total scores range from 7 to 35. The higher the score is, the better the tutors-postgraduates’ interactions are. In the present study, Cronbach’s alpha coefficient of these two subscales are 0.905, and 0.963, respectively.

#### Research training environment.

Postgraduates’ research training environment was measured by using the subscales of the “Postgraduate Research Experience Survey (PRES) 2023”, which was designed by UCL Doctoral School [47]. Three subscales were used: resource (seven items, i.e., “I have a suitable working space when I am studying remotely”), research culture (four items, i.e., “The research community in my research area influences my work”), and community (three items, i.e., “I feel a sense of belonging at my institution”). Responses are made on a 5-point Likert scale, ranging from 1 = definitely disagree to 5 = definitely agree. The subscales’ total scores are 7–35, 4–20, and 3–15, respectively. The higher the score is, the better the research training environment is. In the present study, Cronbach’s alpha coefficient of the three subscales are 0.961, 0.932, and 0.931, respectively.

#### Academic self-efficacy.

Academic self-efficacy was measured by using the self-efficacy questionnaire [48], which is single-dimensional and includes three questions (i.e., “I am confident that I can do scientific research”). Responses are rated on a 6-point Likert scale that ranged from 1 = strongly disagree to 6 = strongly agree. The total scores range from 3 to 18, with higher scores indicating greater academic self-efficacy. This measure has demonstrated good reliability among postgraduates in China [49]. The Cronbach’s alpha coefficient for this study is 0.973.

#### General psychological distress (depression, anxiety, and stress).

The Depression Anxiety Stress Scales 21 (DASS-21) was used to measure general psychological distress in this study. It is a short form of Lovibond and Lovibond’s 42-item self-report measure of depression, anxiety, and stress (DASS) [50]. The questionnaire has 21 questions and includes three subscales: depression (seven items, i.e., “I felt downhearted and blue”), anxiety (seven items, i.e., “I was worried about situations in which I might panic and make a fool of myself”) and stress (seven items, i.e., “I felt that I was using a lot of nervous energy”). Responses are made on a 4-point Likert scale, ranging from 0 = did not apply to me at all to 3 = applied to me very much or most of the time. The maximum score for each scale is 21. The higher scores indicate higher levels of depression, anxiety, or stress. This measure has demonstrated good reliability among different Chinese populations [51]. In the present study, of Cronbach’s alpha coefficient of the three subscales (depression, anxiety, and stress) were 0.936, 0.916, and 0.934, respectively.

#### Fear of future violence at work.

Fear of future violence at work was measured by using the Fear of Future Violence at Work (FFVW) scale, which was designed by Schsat and Kelloway [52]. This 12-item scale was used to assess the degree to which individuals were afraid of experiencing violence at work during the next year (i.e., “I am afraid that I will be sworn at while I’m at work”). Responses are rated on a 7-point Likert scale that ranged from 1 = strongly disagree to 7 = strongly agree. The total FFVW score ranged from 12 to 84, which was divided into three score segments: 12–36 (low degree of fear), 37–60 (medium degree of fear), and 61–84 (high degree of fear). The scale has been demonstrated highly reliable among nurses in China [53]. Cronbach’s alpha coefficient for the FFVW scores in this study was 0.982.

#### Dropout intention.

Dropout intention was assessed based on a question: “Have you ever thought about dropping out of medical school during graduate school?” The response had two options (“yes” or “no”). The selection of this item intends to filter students who considered the possibility of dropping out from medical school independently of the level of seriousness of the thoughts [16].

### Statistical analyses

Data were analyzed using IBM SPSS Statistics Version 27.0 [IBM, 2020]. Firstly, the characteristics of the overall sample were analyzed by descriptive statistics. For the purpose of describing statistics, continuous variables were presented as means ± SD for normally distributed data, while presented as medians and interquartile ranges (1st quartile, 3rd quartile) for non-normally distributed data. Secondly, descriptive statistics and bivariate analysis of sample characteristics for ST and NST groups were analyzed, respectively. Specifically, Pearson’s chi-squared test or Fisher’s exact test was used for analyzing the association between independent and dependent variables at 5% level of signiﬁcance. Finally, the forward multivariate stepwise logistic regression was used to assess the association of demographic characteristics, TPI, RTE, general psychological distress (i.e., depression, anxiety, and stress), academic self-efficacy, and FFVW with dropout intention, and the strength of association was assessed using Odds Ratio (OR). The Hosmer-Lemeshow goodness-of-fit test was used to evaluate the multivariable model fit. We have checked all the assumptions of logistic regression. All the tests were two-tailed, with *P* < 0.05 indicating statistical significance.

## Results

### Demographic characteristics

Participants’ demographic information is shown in S1 Table of the supplementary material. Through screening for usable data, a total of 1,042 participants were included in the final analysis. In terms of gender distribution, more than half of the participants were females (67.2%). The majority of participants were under 25 years of age (74.8%), and the largest proportion (83.4%) was in the first and second grade master. Participants’ grades were predominantly in the middle third of the class (42.5%), and more than half of the participants were from rural (57.9%) and non-only-child families (62.8%). In addition, the educational level of participants’ parents was mainly junior high school or below (father: 52.6%, mother: 62.2%).

A comparison of demographic characteristics of medical college postgraduates who receive ST and NST is also shown in S1 Table of the supplementary material. The results showed that 485 (46.5%) postgraduates were classified as the ST group, and the remaining 557 (53.5%) postgraduates were classified as the NST group. The prevalence of dropout intention was 13.0% for postgraduates in the ST group and 19.6% for postgraduates in the NST group. The two groups had statistical differences in grade, source of postgraduates, one-child households, TPI (comprehensive cultivation interaction), RTE (research culture), and FFVW (*P* < 0.05). Other demographic variables did not reach a statistically significant difference between the two groups (*P* > 0.05).

### Bivariate analysis of dropout intention among medical college postgraduates who receive ST and NST

The study evaluated possible factors associated with the dropout intention of medical college postgraduates who receive ST (see S2 Table of the supplementary material) and NST (see S3 Table of the supplementary material). The possible factors include demographic characteristics, TPI, RTE, general psychological distress (i.e., depression, anxiety, and stress), academic self-efficacy, and FFVW.

For postgraduates in the ST group (see S2 Table of the supplementary material), no significant association was found between their dropout intention and demographic characteristics (*P* > 0.05). While, their dropout intention was associated with satisfaction with the experience of the research degree program (*P* < 0.001), TPI (*P* < 0.001), RTE (*P* < 0.001), general psychological distress (*P* < 0.001), academic self-efficacy (*P* < 0.001), and FFVW (*P* < 0.001).

For postgraduates in the NST group (see S3 Table of the supplementary material), their dropout intention was associated with grade and one-child households (*P* < 0.001). The associations between their dropout intention and other demographic variables were not statistically significant (*P* > 0.05). In addition, their dropout intention associated with satisfaction with the experience of the research degree program (*P* < 0.001), TPI (*P* < 0.001), RTE (*P* < 0.001), general psychological distress (*P* < 0.001), and academic self-efficacy (*P* < 0.001), while their dropout intention was not associated with FFVW (*P* > 0.05).

### Multivariate stepwise logistic regression analysis of dropout intention among medical college postgraduates

Multivariate stepwise logistic regression analysis was used to examine the associations among demographic characteristics, TPI, RTE, general psychological distress, academic self-efficacy and FFVW with dropout intention of medical college postgraduates who receive ST and NST, respectively, and analysis results are presented in Table 1 and 2, respectively.

**Table 1:** Logistic regression analysis of factors associated with DI among medical college postgraduates who receive ST (n = 485).

Variables	b	S_b_	Wald χ^2^	*P* value	OR	OR 95%CI	
LL	UL
Age(Ref: ≤ 25)			5.281	0.071			
26-30	0.293	0.357	0.675	0.411	1.341	0.666	2.701
≥31	0.553	0.453	1.492	**0.025**	**5.110**	**1.226**	**21.301**
**TPI**							
Professional ability interaction	0.111	0.045	6.132	**0.013**	**1.118**	**1.023**	**1.220**
**RTE**							
Community	−0.370	0.094	15.333	**<0.001**	**0.691**	**0.574**	**0.831**
**Academic self-efficacy**	−0.315	0.071	19.464	**<0.001**	**0.730**	**0.635**	**0.840**
**FFVW(Ref:low)**							
Median	0.809	0.397	4.156	**0.041**	**2.245**	**1.032**	**4.886**
High	1.024	0.466	4.831	**0.028**	**2.783**	**1.117**	**6.933**
**Constant**	2.353	1.141	4.254	0.039	10.514		

Notes: TPI = “Tutor-Postgraduate Interaction”; RTE = “Research Training Environment”; FFVW = “Fear of Future Violence at Work”.

**Table 2:** Logistic regression analysis of factors associated with DI among medical college postgraduates who receive NST (n = 557).

Variables	b	S_b_	Wald χ^2^	*P* value	OR	OR 95%CI	
LL	UL
**Father’s education level** (**Ref:** Junior high school or below)			6.048	0.049			
Senior high school (or technical secondary school)	0.728	0.297	6.020	**0.014**	**2.072**	**1.158**	**3.708**
College or above (include junior college)	0.258	0.380	0.460	0.498	1.294	0.614	2.725
**Satisfaction with the experience of research degree program** (**Ref:** Very dissatisfied)							
Not satisfied	−0.795	0.764	1.083	0.298	0.452	0.101	2.018
Neutral	−2.227	0.675	10.890	**<0.001**	**0.108**	**0.029**	**0.405**
Satisfied	−2.901	0.697	17.340	**<0.001**	**0.055**	**0.014**	**0.215**
Very satisfied	−3.459	0.895	14.950	**<0.001**	**0.031**	**0.005**	**0.182**
**TPI**							
Professional ability interaction	0.065	0.044	2.173	0.140	1.067	0.979	1.163
Comprehensive cultivation interaction	−0.068	0.033	4.190	**0.041**	**0.935**	**0.876**	**0.997**
**General psychological distress**							
Stress	0.118	0.030	15.909	**<0.001**	**1.125**	**1.062**	**1.192**
**Academic self-efficacy**	−0.143	0.048	8.726	**0.003**	**0.867**	**0.788**	**0.953**
**Constant**	0.904	1.312	0.475	0.491	2.470		

Notes: TPI = “Tutor-Postgraduate Interaction”.

The results indicated that age, TPI (professional ability interaction), and FFVW are risk factors for dropout intention of postgraduates who receive ST (see Table 1). Specifically, those over the age of 31 (OR = 5.110, 95%CI: 1.226–21.301, *P* = 0.025) have 5.110 times greater chance of exhibiting dropout intention when compared with those under the age of 25; those with medium(OR = 2.245, 95%CI: 1.032–4.886, *P* = 0.041) and high(OR = 2.783, 95%CI: 1.117–6.933, *P* = 0.028) levels of FFVW have 2.245 and 2.783 times greater chance of exhibiting dropout intention respectively when compared with those with low levels of FFVW. Additionally, the results indicated that community (OR = 0.691, 95%CI: 0.574–0.831, *P* < 0.001) and academic self-efficacy (OR = 0.730, 95%CI: 0.635–0.840, *P* < 0.001) are protective factors for dropout intention of postgraduates who receiving ST. Adequate calibration was assessed by the Hosmer-Lemeshow test (χ^2^ = 5.260, *P* = 0.729).

The results indicated that the father’s education level and stress are risk factors for the dropout intention of medical college postgraduates who receive NST (see Table 2). Medical college NST postgraduates whose fathers with a senior high school education level have 2.072 times (OR = 2.072, 95%CI: 1.158–3.708, *P* = 0.014) greater chance of dropping out than those whose fathers’ education level is Junior high school or below; the higher level of stress have 1.125 times (OR = 1.125, 95%CI: 1.062–1.192, *P* < 0.001) greater chance of exhibiting dropout intention when compared with lower level.

Additionally, the results indicated that TPI (comprehensive cultivation interaction) (OR = 0.935, 95%CI: 0.876 ~ 0.997, *P* = 0.041) and academic self-efficacy (OR = 0.867, 95%CI: 0.788 ~ 0.953, *P* = 0.003) were found to be protective factors for dropout intention. The greater TPI (comprehensive cultivation interaction) and academic self-efficacy are associated with a lower level of dropout intention. Satisfaction with the experience of the research degree program with “Neutral”(OR = 0.108, 95%CI: 0.029–0.405, *P* < 0.001), “Satisfied” (OR = 0.055, 95%CI: 0.014–0.215, *P* < 0.001) and “Very satisfied” (OR = 0.031, 95%CI: 0.005–0.182, *P* < 0.001) medical college postgraduates receiving NST had a lower likelihood of dropout intention when compared with whose satisfaction with “Very dissatisfied”, indicating that the satisfaction with the experience of the research degree program is a protective factor against dropout intention. Adequate calibration was assessed by the Hosmer-Lemeshow test (χ^2^ = 8.076, *P* = 0.426).

## Discussion

This study was the first to explore the potential similarities and differences in factors influencing the dropout intention of medical college postgraduates who received ST and NST in China. Factors influencing dropout intention differ among postgraduates in different nurturing environments. Additionally, this study was the first to investigate the dropout intention rates for both groups. The results showed that 16.5% of medical college postgraduates had an intention to drop out, with 13.0% among those in ST and 19.6% among those in NST expressing this intention.

### Factors related to the dropout intention of medical college postgraduates who receive ST

#### The risk factors.

This study found a significant association between age and dropout intention in medical college postgraduates who received ST. Older postgraduates exhibited a higher propensity to drop out than their younger counterparts, which is consistent with previous studies [54]. The possible explanation is that, self-determined motivation and the willingness to pursue a medical career decline with increased age, due to the doctor-patient relationship or burnout [55].

Additionally, TPI (professional competence interaction) proved to be a risk factor for the dropout intention of postgraduates who received ST, which is inconsistent with previous studies [56,57]. There are three potential explanations for this finding. First, standardized training represents the transition period from being a medical student to becoming a junior doctor and it typically entails considerable stress [58]. During the standardized training year, medical students are assumed to take responsibility for primary care in inpatient services and are frequently on call for night duty. This not only increases responsibility for patient care and the long working hours, also increases the stress level for postgraduates who receive ST [59]. Furthermore, professional interactions may exacerbate academic pressure for ST postgraduates, potentially contributing to attrition. Palička et al. found that the vast majority of Czech medical students experience excessive stress during their studies, and students experiencing higher levels of excessive stress are more likely to leave their studies based on their own decisions [60]. Second, from the tutors’ perspective, the contractual mentoring relationship tends to overemphasize instrumental rationality in research activities while undervaluing academic spirit and humanistic values. This approach disregards postgraduate students’ autonomy, thereby weakening the nurturing dimension of mentorship. Consequently, the supervisor-student relationship often becomes instrumental and impersonal, resembling an employment contract rather than an academic guidance relationship [61]. Third, from the postgraduates’ perspective, pursuing advanced studies is primarily motivated by employment prospects or other utilitarian objectives rather than purely academic interests. Consequently, their academic commitment remains weak, often resulting in a perfunctory approach to research that deviates from the truth-seeking essence of scientific inquiry. Under such circumstances, tutor-postgraduate interaction may inadvertently exacerbate students’ dropout intentions [62].

Furthermore, the study found that medium and high levels of FFVW significantly increase dropout intention among postgraduates who received ST in medical college compared to low levels of FFVW. The incidence of workplace violence against medical interns is notably high [63], which may hinder medical students’ career planning and future retention rates [64]. In Chile, 91% of medical students reported experiencing at least one abusive episode during their training, with 32% considering dropping out as a result [65]. Medical colleges should closely monitor internship environments to prevent workplace violence and provide psychological counseling to interns.

### The protective factors

This study found that a sense of community belonging in the research training environment positively influenced the dropout intention of postgraduates who received ST in this study, which is consistent with previous studies [35]. Feelings of belonging imply that the postgraduates feel valuable and respected in their educational program [66,67]. When respect is lacking, there is a greater vulnerability to academic failure and may lead to an increasing likelihood of dropping out [38]. According to the theory of school membership [68], the research training environment and the sense of belonging are critical for students’ emotional resilience in dealing with difficult emotions [35]. Students are better able to integrate into research training environments, form positive relationships, and generate positive emotions when they have a higher sense of belonging and cohesion with the community [69]. The combination of school belonging and educational commitment promotes more positive academic and social interactions [70].

### Factors related to dropout intention of medical college postgraduates who receive NST

#### The risk factors.

This study found that a higher level of father’s education was a risk factor for dropout intention among medical college postgraduates who received NST, which is consistent with previous studies. For example, Truta et al. (2018) [26] found that students from families with low education levels reported higher levels of academic engagement, which can prevent dropout intention [16]. More than half of the postgraduates who received NST come from rural areas, where their fathers usually have lower education levels (also can be seen in our data). Their families may be less affluent compared to those with highly educated parents. As the Chinese saying goes, “Social Mobility of Disadvantaged”, these students may be more motivated to change their lives through their efforts and are therefore less likely to drop out.

Additionally, a higher level of general psychological distress (stress) was associated with a higher level of dropout intention among medical college postgraduates who received NST. Palička et al. (2023) [60] found that the vast majority of Czech medical students experience excessive stress during their studies, and those who experience higher levels of stress are more likely to leave their studies voluntarily. Medical students often face pressures from various sources during their postgraduate studies [71], which not only impacts students’ mental health but also results in high rates of medical school dropout [72]. Psychological Counseling Center of medical colleges should assess and prevent stress timely, and conduct stress prevention training to reduce dropout intention among medical college postgraduates.

### The protective factors

TPI (comprehensive cultivation interaction) between tutors and postgraduates has been identified as a protective factor for dropout intention in postgraduates who received NST. The influence of tutors on postgraduates plays an irreplaceable role in their mental health education [73]. According to social cognitive theory [74], tutor-postgraduate rapport influences graduate students’ self-efficacy [75]. Tutors can improve postgraduates’ self-efficacy to combat psychological stress and improve retention in academic careers [76]. Medical colleges should strengthen the theoretical and practical training of tutors in educational psychology to improve the level of instruction and alleviate postgraduates’ emotions.

Higher satisfaction with the research degree program among medical college postgraduates who received NST was associated with lower dropout intention. According to Ecological systems theory [77], students’ development is influenced by various factors, such as the research training environment, tutors’ mentoring approach, and academic engagement. These factors may affect satisfaction with the research degree program through research experience. Just as job satisfaction influences the propensity for academic brain drain [78], academic satisfaction is a significant predictor of students’ dropout intention [27]. Providing good educational support in schools positively impacts students’ academic career satisfaction to some extent [79].

### Differences and similarities in factors related to dropout intention of medical college postgraduates who receive ST and NST.

Our study also found several interesting results. For the ST group, age, TPI (professional ability interaction), and FFVW are risk factors related to dropout intention, while community and academic self-efficacy are protective factors related to dropout intention. For the NST group, the father’s education level and stress are risk factors related to dropout intention, while TPI (comprehensive cultivation interaction), academic self-efficacy and satisfaction with the experience of the research degree program are protective factors related to dropout intention.

In terms of demographics, age was a risk factor for the dropout intention of postgraduates who received ST in medical college [54], but it had no associations with the dropout intention of postgraduates who received NST. In China, postgraduates who receive ST are better positioned for career development compared to those who receive NST. However, older postgraduates who receive ST are less conducive to career development, resulting in a higher likelihood of dropout compared to their younger counterparts. Additionally, a father’s education level was identified as a risk factor for dropout intention in NST groups [26], but it had no association with dropout intention for ST groups.

Due to distinct training content and environment between ST and NST groups, several influencing factors differ. In terms of TPI, postgraduates who receive ST face a large amount of clinical work and TPI (professional competence interaction), which increases their stress and burnout. Postgraduates who receive NST experience greater academic pressure in the process of scientific research, so the TPI (comprehensive cultivation interaction) reduces the willingness of postgraduates who receive NST to drop out. In terms of FFVW, the ST group exposed to real clinical settings are more likely to experience workplace violence such as medical troubles and doctor-patient relationships when compared with the NST group. In the community context, postgraduates who receive ST require more collective support to overcome the dual challenges of clinical work and scientific research, whereas postgraduates who receive NST tend to conduct scientific research independently and rely less on the group. As for satisfaction with the experience of the research degree program, the NST group holds higher expectations for academic training, while the ST group may prioritize clinical expertise and skills.

Stress was identified as a risk factor for postgraduates who received NST, while it did not exert a statistically significant influence on the dropout intention of postgraduates who received ST. One possible explanation for this discrepancy lies in postgraduates who receive ST have the opportunity to interact with colleagues and be exposed to a diverse group of individuals, which may mitigate their psychological stress through a supportive group atmosphere. In contrast, postgraduates who receive NST encounter higher academic expectations and limited opportunities for social interaction, which may lead to greater academic pressure.

Research self-efficacy serves as a protective factor against dropout intention for medical college postgraduates who receive both ST and NST, which is consistent with previous studies. Despite differing training focuses, the development of research skills is essential for both types of master’s degree education [80]. Academic self-efficacy is a key indicator of students’ psychological well-being and a positive psychological variable in preventing academic burnout and dropout [81]. Bierer et al. (2015) [82] found that medical students with high research self-efficacy are more likely to pursue careers related to their clinical research interests. Academic self-efficacy positively predicts academic engagement [83], which serves as a protective factor against dropout intention [84]. Previous studies have shown that positive supervisor–student relationships promote students’ self-efficacy [49]. Therefore, medical colleges should actively promote mentoring interactions to create a supportive research environment for postgraduates.

To minimize the negative impact of dropout intention on graduate students and reduce the dropout rate, the following recommendations could be provided based on the findings of this study. Firstly, the medical colleges should establish distinct training strategies tailored to the different environments of postgraduates in ST and NST. For postgraduates who receive ST, the medical colleges should work with hospitals to create rationalized training plans, enhance emotional connections among colleagues, foster a harmonious team atmosphere, optimize hospital management, and reduce workplace violence. Additionally, medical colleges should provide regular psychological counseling to reduce postgraduates’ fear of workplace violence and promote their mental health, with particular attention to the psychological health of older postgraduates. For postgraduates who receive NST, a scientific tutor training system should be established by the medical colleges, incorporating professional development and ethical education for tutors. Additionally, the assessment of tutors’ mental health education should be strengthened. A Mental Health Advisory Committee should be established to monitor the mental health of students regularly.

### Limitations

This study explored the factors related to dropout intention among Chinese medical college postgraduates, including ST and NST groups. While this study presents valuable findings, it is necessary to acknowledge some limitations that need to be further improved in future studies. Firstly, the cross-sectional approach used in this study could not establish causal relationships. Future research could further investigate the causal relationships between variables through cohort studies. Secondly, the use of convenience sampling in this study may have caused bias, and in the future it is recommended that other sampling methods be used to reduce the impact of bias on the results of the study. Thirdly, all variables were measured using a self-reporting method, which may cause reporting bias. Therefore, it is suggested that future studies should employ more objective methodologies to measure these variables. Finally, the study sample consisted of medical students from a single medical school in China, which may lack of representativeness. Future studies should include postgraduates from various regional medical universities in China to enhance generalizability.

## Conclusion

This study has two contributions to the research. Firstly, it provided up-to-date data on the proportion of dropout intention among medical college postgraduates in China. Secondly, it showed multiple factors that related to their dropout intention. Specifically, it investigated the factors related to dropout intention in two distinct groups: medical postgraduates who received ST and NST. And both risk and protective factors associated with dropout intention among medical college postgraduates who receiving ST and NST were found. It further explored the potential similarities and differences of factors related to dropout intention between these groups. Future research could utilize the findings of this study to propose more targeted strategies aimed at addressing these risk and protective factors, thereby reducing dropout intention and rates and fostering stable and healthy development within the medical workforce.

## Supporting information

S1 Table S1 Table of the supplementary material. (DOCX)

S2 Table S2 Table of the supplementary material. (DOCX)

S3 Table S3 Table of the supplementary material. (DOCX)

## Abbreviations:

ST: Standardized Training
NST: Non-Standardized Training
DI: Dropout Intention
NDI: Non-Dropout Intention
TPI: Tutors-Postgraduate Interaction
RTE: Research Training Environment
FFVW: Fear of Future Violence at Work
