# Supplementary material for: Factors related to dropout intention of medical college postgraduates in China: A comparison between students who receive standardized training and non-standardized training
Source: PLoS One. 2025 Jul 2;20(7):e0325146. doi: 10.1371/journal.pone.0325146 (PMC12221019; doi:10.1371/journal.pone.0325146)
Supplement: S1 Table — (DOCX) [file pone.0325146.s001.docx]

**Table 1. Comparison of demographic characteristics between medical college postgraduates who receive ST and NST**

| **Variables** | **Overall sample** | **ST** | **NST** | **χ²*/Z*** | ***P*** |
| --- | --- | --- | --- | --- | --- |
|  | **N = 1042 (100%)** | **n = 485 (46.5%)** | **n = 557 (53.5%)** |  |  |
|  |  |  |  |  |  |
| **DI** |  |  |  | **8.143** | **0.004** |
| Yes | 172 (16.5%) | 63 (13.0%) | 109 (19.6%) |  |  |
| No | 870 (83.5%) | 422 (87.0%) | 448 (80.4%) |  |  |
| **Gender** |  |  |  | 1.069 | 0.301 |
| Male | 342 (32.8%) | 167 (34.4%) | 175 (31.4%) |  |  |
| Female | 700 (67.2%) | 318 (65.6%) | 382 (68.6%) |  |  |
| **Age** |  |  |  | 4.589 | 0.101 |
| ≤25 | 779 (74.8%) | 367 (75.7%) | 412 (74.0%) |  |  |
| 26-30 | 223 (21.4%) | 106 (21.9%) | 117 (21.0%) |  |  |
| ≥31 | 40 (3.8%) | 12 (2.5%) | 28 (5.0%) |  |  |
| **Grade** |  |  |  | **8.12** | **0.017** |
| First Grade Master | 578 (55.5%) | 285 (58.8%) | 293 (52.6%) |  |  |
| Second Grade Master | 291 (27.9%) | 136 (28.0%) | 155 (27.8%) |  |  |
| Third Grade Master | 173 (16.6%) | 64 (13.2%) | 109 (19.6%) |  |  |
| **Academic performance** |  |  |  | 2.164 | 0.339 |
| The first third | 356 (34.2%) | 157 (32.4%) | 199 (35.7%) |  |  |
| The middle third | 443 (42.5%) | 206 (42.5%) | 237 (42.5%) |  |  |
| The last third | 243 (23.3%) | 122 (25.2%) | 121 (21.7%) |  |  |
| **Source of students** |  |  |  | **7.891** | **0.005** |
| Urban | 439 (42.1%) | 182 (37.5%) | 257 (46.1%) |  |  |
| Rural | 603 (57.9%) | 303 (62.5%) | 300 (53.9%) |  |  |
| **One-child households** |  |  |  | **4.014** | **0.045** |
| Yes | 388 (37.2%) | 165 (34.0%) | 223 (40.0%) |  |  |
| No | 654 (62.8%) | 320 (66.0%) | 334 (60.0%) |  |  |
| **Father’s education level** |  |  |  | 2.006 | 0.367 |
| Junior high school or below | 548 (52.6%) | 262 (54.0%) | 286 (51.3%) |  |  |
| Senior high school | 308 (29.6%) | 133 (27.4%) | 175 (31.4%) |  |  |
| (or technical secondary school) |  |  |  |  |  |
| College or above | 186 (17.9%) | 90 (18.6%) | 96 (17.2%) |  |  |
| （including junior college） |  |  |  |  |  |
| **Mother’s education level** |  |  |  | 3.514 | 0.173 |
| Junior high school or below | 648 (62.2%) | 311 (64.1%) | 337 (60.5%) |  |  |
| Senior high school | 251 (24.1%) | 104 (21.4%) | 147 (26.4%) |  |  |
| (or technical secondary school) |  |  |  |  |  |
| College or above | 143 (13.7%) | 70 (14.4%) | 73 (13.1%) |  |  |
| （including junior college） |  |  |  |  |  |
| **Satisfaction with the experience of** |  |  |  | 4.315 | 0.365 |
| **research degree program** |  |  |  |  |  |
| Very dissatisfied | 28 (2.7%) | 9 (1.9%) | 19 (3.4%) |  |  |
| Not satisfied | 73 (7.0%) | 35 (7.2%) | 38 (6.8%) |  |  |
| Neutral | 369 (35.4%) | 163 (33.6%) | 206 (37.0%) |  |  |
| Satisfied | 403 (38.7%) | 194 (40.0%) | 209 (37.5%) |  |  |
| Very satisfied | 169 (16.2%) | 84 (17.3%) | 85 (15.3%) |  |  |
| **TPI** |  |  |  |  |  |
| Professional ability interaction score, median (IQR) | 28 (27,32) | 28 (27,33) | 28 (27,32) | -1.366 | 0.172 |
| Comprehensive cultivation interaction score, median (IQR) | 28 (25,35) | 28 (26,35) | 28 (25,34) | **-2.228** | **0.026** |
| **RTE** |  |  |  |  |  |
| Resource score, median (IQR) | 28 (26,35) | 28 (26,35) | 28 (26,34) | -0.936 | 0.349 |
| Research Culture score, median (IQR) | 16 (14,20) | 16 (14.5,20) | 16 (14,18) | **-2.014** | **0.044** |
| Community score, median (IQR) | 12 (11,15) | 12 (11,15) | 12 (11,15) | -1.313 | 0.189 |
| **General psychological distress** |  |  |  |  |  |
| Depression score, median (IQR) | 8 (7,13) | 8 (7,13) | 9 (7,13) | -1.021 | 0.307 |
| Anxiety score, median (IQR) | 8 (7,12) | 8 (7,12) | 8 (7,13) | -1.314 | 0.189 |
| Stress score, median (IQR) | 9 (7,14) | 9 (7,14) | 9 (7,14) | -1.66 | 0.097 |
| **Academic self-efficacy** |  |  |  |  |  |
| score, median (IQR) | 13 (12,15) | 12 (12,15) | 13 (12,15) | -0.453 | 0.65 |
| **FFVW** |  |  |  |  |  |
| score, median (IQR) | 39 (21,55) | 43 (23.5,58) | 35 (20.5,52.5) | **-2.151** | **0.032** |

Notes: ST = “Standardized Training”; DI = “Dropout Intention”; TPI = “Tutor-Postgraduate Interaction”; RTE= “Research Training Environment”; FFVW = “Fear of Future Violence at Work”.
