# Supplementary material for: Factors related to dropout intention of medical college postgraduates in China: A comparison between students who receive standardized training and non-standardized training
Source: PLoS One. 2025 Jul 2;20(7):e0325146. doi: 10.1371/journal.pone.0325146 (PMC12221019; doi:10.1371/journal.pone.0325146)
Supplement: S2 Table — (DOCX) [file pone.0325146.s002.docx]

**Table 2. Possible factors associated with dropout intention among medical college postgraduates who receive ST (n=485)**

| **Variables** | **DI** | **NDI** | ***P*** |
| --- | --- | --- | --- |
|  | **n = 63 (13.0%)** | **n = 422 (87.0%)** |  |
| **Gender** |  |  | 0.631 |
| Male | 20 (31.7%) | 147 (34.8%) |  |
| Female | 43 (68.3%) | 275 (65.2%) |  |
| **Age** |  |  | 0.055 |
| ≤25 | 42 (66.7%) | 325 (77.0%) |  |
| 26-30 | 17 (27.0%) | 89 (21.1%) |  |
| ≥31 | 4 (6.3%) | 8 (1.9%) |  |
| **Grade** |  |  | 0.14 |
| First Grade Master | 30 (47.6%) | 255 (60.4%) |  |
| Second Grade Master | 26 (41.3%) | 110 (26.1%) |  |
| Third Grade Master | 7 (11.1%) | 57 (13.5%) |  |
| **Academic performance** |  |  | 0.636 |
| The first third | 18 (28.6%) | 139 (32.9%) |  |
| The middle third | 29 (46.0%) | 177 (41.9%) |  |
| The last third | 16 (25.4%) | 106 (25.1%) |  |
| **Source of students** |  |  | 0.116 |
| Urban | 18 (28.6%) | 164 (38.9%) |  |
| Rural | 45 (71.4%) | 258 (61.1%) |  |
| **One-child households** |  |  | 0.902 |
| Yes | 21 (33.3%) | 144 (34.1%) |  |
| No | 42 (66.7%) | 278 (65.9%) |  |
| **Father’s education level** |  |  | 0.056 |
| Junior high school or below | 41 (65.1%) | 221 (52.4%) |  |
| Senior high school (or technical secondary school) | 14 (22.2%) | 119 (28.2%) |  |
| College or above (including junior college) | 8 (12.7%) | 82 (19.4%) |  |
| **Mother’s education level** |  |  | 0.235 |
| Junior high school or below | 45 (71.4%) | 266 (63.0%) |  |
| Senior high school (or technical secondary school) | 10 (15.9%) | 94 (22.3%) |  |
| College or above (including junior college) | 8 (12.7%) | 62 (14.7%) |  |
| **Satisfaction with the experience of research degree program** |  |  | **<0.001** |
| Very dissatisfied | 4 (6.3%) | 5 (1.2%) |  |
| Not satisfied | 12 (19.0%) | 23 (5.5%) |  |
| Neutral | 33 (52.4%) | 130 (30.8%) |  |
| Satisfied | 11 (17.5%) | 183 (43.4%) |  |
| Very satisfied | 3 (4.8%) | 81 (19.2%) |  |
| **TPI** |  |  |  |
| Professional ability interaction score, median (IQR) | 28 (25,30) | 29 (27,33.25) | **<0.001** |
| Comprehensive cultivation interaction score, median (IQR) | 27 (21,28) | 28 (27,35) | **<0.001** |
| **RTE** |  |  |  |
| Resource score, median (IQR) | 26 (214,28) | 28 (28,35) | **<0.001** |
| Research Culture score, median (IQR) | 14 (12,16) | 16 (15,20) | **<0.001** |
| Community score, median (IQR) | 11 (9,12) | 12 (12,15) | **<0.001** |
| **General psychological distress** |  |  |  |
| Depression score, median (IQR) | 12 (8,16) | 8 (7,12) | **<0.001** |
| Anxiety score, median (IQR) | 11 (7,15) | 8 (7,12) | **<0.001** |
| Stress score, median (IQR) | 14 (8,18) | 8 (7,14) | **<0.001** |
| **Academic self-efficacy** score, median (IQR) | 11 (9,12) | 14 (12,15) | **<0.001** |
| **FFVW** | 51 (41,66) | 39 (19.75,56) | **<0.001** |

Notes: ST = “Standardized Training”; DI = “Dropout Intention”; TPI = “Tutor-Postgraduate Interaction”; RTE= “Research Training Environment”; FFVW = “Fear of Future Violence at Work”.
