# Supplementary material for: Factors related to dropout intention of medical college postgraduates in China: A comparison between students who receive standardized training and non-standardized training
Source: PLoS One. 2025 Jul 2;20(7):e0325146. doi: 10.1371/journal.pone.0325146 (PMC12221019; doi:10.1371/journal.pone.0325146)
Supplement: S3 Table — (DOCX) [file pone.0325146.s003.docx]

**Table 3. Possible factors associated with dropout intention among medical college postgraduates who receive NST (n=557)**

| **Variables** | **DI** | **NDI** | ***P*** |
| --- | --- | --- | --- |
|  | **n=109 (19.6%)** | **n=448 (80.4%)** |  |
| **Gender** |  |  | 0.456 |
| Male | 31 (28.4%) | 144 (32.1%) |  |
| Female | 78 (71.6%) | 304 (67.9%) |  |
| **Age** |  |  | 0.422 |
| ≤25 | 84 (77.1%) | 328 (73.2%) |  |
| 26-30 | 20 (18.3%) | 97 (21.7%) |  |
| ≥31 | 5 (4.6%) | 23 (5.1%) |  |
| **Grade** |  |  | **<0.001** |
| First Grade Master | 38 (34.9%) | 255 (56.9%) |  |
| Second Grade Master | 38 (34.9%) | 117 (26.1%) |  |
| Third Grade Master | 33 (30.3%) | 76 (17.0%) |  |
| **Academic performance** |  |  | 0.564 |
| The first third | 38 (34.9%) | 161 (35.9%) |  |
| The middle third | 53 (48.6%) | 184 (41.1%) |  |
| The last third | 18 (16.5%) | 103 (23.0%) |  |
| **Source of postgraduates** |  |  | 0.715 |
| Urban | 52 (47.7%) | 205 (45.8%) |  |
| Rural | 57 (52.3%) | 243 (54.2%) |  |
| **One-child households** |  |  | **0.020** |
| Yes | 33 (30.3%) | 190 (42.4%) |  |
| No | 76 (69.7%) | 258 (57.6%) |  |
| **Father’s education level** |  |  | 0.275 |
| Junior high school or below | 49 (45.0%) | 237 (52.9%) |  |
| Senior high school (or technical secondary school) | 42 (38.5%) | 133 (29.7%) |  |
| College or above (including junior college) | 18 (16.5%) | 78 (17.4%) |  |
| **Mother’s education level** |  |  | 0.284 |
| Junior high school or below | 60 (55.0%) | 277 (61.8%) |  |
| Senior high school (or technical secondary school) | 35 (32.1%) | 112 (25.0%) |  |
| College or above (including junior college) | 14 (12.8%) | 59 (13.2%) |  |
| **Satisfaction with the experience of research degree program** |  |  | **<0.001** |
| Very dissatisfied | 15 (13.8%) | 4 (0.9%) |  |
| Not satisfied | 26 (23.9%) | 12 (2.7%) |  |
| Neutral | 47 (43.1%) | 159 (35.5%) |  |
| Satisfied | 18 (16.5%) | 191 (42.6%) |  |
| Very satisfied | 3 (2.8%) | 82 (18.3%) |  |
| **TPI** |  |  |  |
| Professional ability interaction score, median (IQR) | 27 (25,30) | 29 (27,32.75) | **<0.001** |
| Comprehensive cultivation interaction score, median (IQR) | 25 (21,28.5) | 28 (26,35) | **<0.001** |
| **RTE** |  |  |  |
| Resource score, median (IQR) | 27 (21.5,28) | 28 (27,35) | **<0.001** |
| Research Culture score, median (IQR) | 14 (12,16) | 16 (15,20) | **<0.001** |
| Community score, median (IQR) | 11 (9,12) | 12 (12,15) | **<0.001** |
| **General psychological distress** |  |  |  |
| Depression score, median (IQR) | 14 (10,18) | 8 (7,11) | **<0.001** |
| Anxiety score, median (IQR) | 13 (9,16) | 8 (7,11) | **<0.001** |
| Stress score, median (IQR) | 15 (11,19) | 9 (7,13) | **<0.001** |
| **Academic self-efficacy** score, median (IQR) | 11 (9,12.5) | 15 (12,15) | **<0.001** |
| **FFVW** | 40 (23.5,54.5) | 34 (19.25,51) | 0.098 |

Notes: DI = “Dropout Intention”; TPI = “Tutor-Postgraduate Interaction”; RTE= “Research Training Environment”; FFVW = “Fear of Future Violence at Work”.
